# Plate size reduction surgery for the Baerveldt 350-mm^2^ glaucoma implant for postoperative motor disturbance: A case report

**DOI:** 10.1097/MD.0000000000017163

**Published:** 2019-09-13

**Authors:** Hirotaka Tanabe, Shunsuke Nakakura, Asuka Noguchi, Hitoshi Tabuchi, Yoshiaki Kiuchi

**Affiliations:** a Department of Ophthalmology, Saneikai Tsukazaki Hospital, Himeji, Hyogo; b Department of Ophthalmology and Visual Sciences, Graduate School of Biomedical Sciences, Hiroshima University, Hiroshima, Japan.

**Keywords:** Baerveldt, diplopia, glaucoma, motor disturbance

## Abstract

**Rationale:**

Diplopia due to ocular motility disturbance is a common complication after glaucoma drainage device (GDD) surgery. The treatment options include prescription prism glasses, strabismus surgery or GDD removal. However, to the best of our knowledge, GDD size reduction surgery has not been reported.

**Patient concerns and diagnoses:**

An 83-year-old woman diagnosed with primary open angle glaucoma was referred to Tsukazaki Hospital due to uncontrolled intraocular pressure (IOP) in December 2015. We performed an EXPRESS shunt surgery on both eyes in January 2016 and a needling procedure on the left eye in May 2017. Thereafter, because IOP in her left eye remained high, we performed Baerveldt 350-mm^2^ implantation in her inferotemporal area by placing the tube at the sulcus on December 3, 2017. The next day, 4Δ hypertropia (HT) was detected in the left eye in alternate cover testing in primary gaze, and diplopia in the inferotemporal direction was demonstrated. Although IOP was controlled well between 15 and 20 mmHg in her left eye, diplopia did not improve.

**Interventions:**

Three weeks later, we performed a plate size reduction surgery for the Baerveldt 350-mm^2^ implant. In this procedure, we cut and removed the plates placed beneath the lateral rectus muscle and inferior rectus muscle, which were thought to be responsible for diplopia.

**Outcomes:**

Diplopia improved subjectively, but there was no drastic objective change. We prescribed prism glasses (3Δ base down for the left eye) for remaining mild diplopia. On January 21, 2019, significant objective improvement (2Δ HT with less ocular motor dysfunction demonstrated in the Hess chart) was finally observed.

**Lessons:**

Early plate size reduction surgery, which was not immediately but ultimately effective in improving motor disturbance in our case, could be a potential option to relieve operation-induced motor disturbance. However, notably, tube shunt surgery has the risk of motility disturbances, which might require additional treatment.

## 1 Introduction

Diplopia due to ocular motility disturbance is a common complication after glaucoma drainage device (GDD) surgery. The Baerveldt implant is a major GDD device that is effective in controlling intraocular pressure (IOP) because of the greater surface area for passive diffusion. However, the incidence of persistent postoperative strabismus associated with the Baerveldt implant ranges from 2.1% to 77% and that of diplopia from 1.4 to 37% in case series.^[1–7]^ In a prospective randomized clinical trial, the incidence of postoperative strabismus associated with the Baerveldt implant was 9.9% and that of diplopia was 5% in the Tube vs trabeculectomy (TVT) study.^[8]^ Additionally, the incidence of diplopia was 2.8% in the Ahmed Baerveldt Comparison (ABC) study.^[9]^ The hypothesized cause of diplopia is mechanical disturbance attributable to the size of the plate, size of the resultant bleb and postoperative adhesion of the surrounding tissue of the rectus muscles.^[10–13]^ The treatment options for postoperative diplopia after GDD surgery include prescription prism glasses, strabismus surgery or GDD removal. However, to the best of our knowledge, GDD size reduction surgery has not been reported. The present report describes a plate size reduction surgery for a Baerveldt 350-mm^2^ glaucoma implant performed in a patient who experienced postoperative ocular motility disturbance after Baerveldt 350-mm^2^ implantation surgery. Additionally, we reviewed previous reports on treatment of diplopia induced by glaucoma implant surgery. This case report was approved by the Research Ethics Committee of Saneikai Tsukazaki Hospital (Himeji, Japan), and the patient provided informed consent for publication of the case.

## 2 Case report

**Figure 1:**
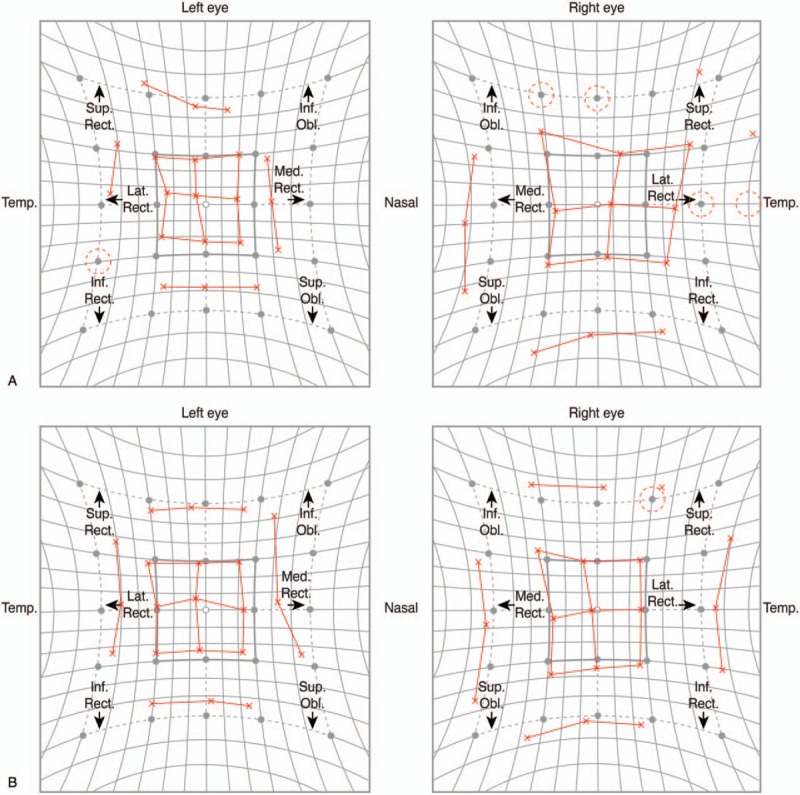
A, B. Hess charts performed 3 weeks after the Baerveldt 350-mm^2^ implantation (A) and approximately one year after the plate size reduction surgery (B) are shown.

**Figure 2:**
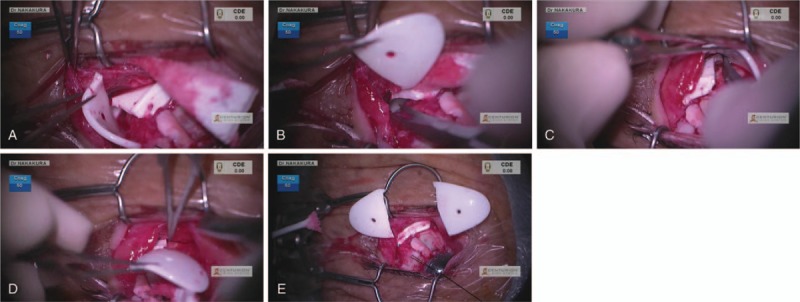
A–E. We cut and partially removed the Baerveldt 350-mm^2^ glaucoma implant of which 2 plate parts were placed beneath the lateral rectus muscle (A, B) and inferior rectus muscle (C, D), which were thought to be responsible for diplopia. A postresection image is also shown (E).

An 83-year-old woman diagnosed with primary open angle glaucoma was referred to Tsukazaki Hospital due to uncontrolled ocular pressure in December 2015. At the first visit, the IOP was 28 mmHg in both eyes under maximum anti-glaucoma eyedrop treatment. The mean deviation given by Humphrey Visual Field Analyzer (30–2 program) was −23.86 dB in the right eye and −16.31 dB in the left eye. We performed an EXPRESS shunt surgery on both eyes in the superotemporal area in January 2016 and a needling procedure on the left eye in May 2017. Thereafter, IOP in her left eye remained high (22 mmHg with the use of 2 types of anti-glaucoma eyedrops). Therefore, we performed Baerveldt 350-mm^2^ implantation at her inferotemporal area, placing the tube at the sulcus on December 3, 2017. The next day, 4Δ hypertropia (HT) was detected in alternate cover testing (APCT) with prism measurement in primary gaze, and significant ocular motility disturbance caused diplopia in gaze to the quadrant direction from inferior to temporal in which the Baerveldt 350-mm^2^ was implanted. Although the IOP was controlled well between 15 and 20 mmHg in her left eye, diplopia did not improve for 3 weeks. The Hess chart suggested persistent motor disturbance of both inferotemporal and superonasal directions (Fig. 1A), which could be attributed to the following reasons: motor disturbance in the inferotemporal direction due to the large size of the Baerveldt implant and motor disturbance in the superonasal direction due to muscle contraction of the rectus muscles under which the Baerveldt implant was placed. On December 23, 2017, we performed a plate reduction surgery for the Baerveldt 350-mm^2^ glaucoma implant. In this procedure, we first confirmed the resistance to both inferotemporal and superonasal directions and the absence of apparent adhesions and scar tissues. Then, we cut and removed the plates placed beneath the lateral rectus muscle and inferior rectus muscle, which were thought to be responsible for diplopia (Fig. 2A–E). After the procedure, diplopia improved subjectively, but there was no drastic objective change with remaining 4Δ HT in APCT in primary gaze. We prescribed prism glasses (3Δ base down [BD] for the left eye) for remaining mild diplopia for this patient. Thereafter, IOP was controlled well between 13 and 15 mmHg in her left eye with only topical Dorzolamide 2%. On January 21, 2019, 1 year after the operation, significant objective improvement (2Δ HT in APCT with less ocular motor dysfunction demonstrated in the Hess chart (Fig. 1B)) was finally observed.

## 3 Discussion

We reported the case of a patient who developed postoperative diplopia after undergoing Baerveldt 350-mm^2^ glaucoma implantation surgery wherein an earlier Baerveldt plate reduction surgery was not immediately but ultimately effective in improving motor disturbance. The mechanism of diplopia induced by mechanical disturbance was discussed in detail by Muñoz and Parrish.^[10]^ If a large bleb displaces the muscle away from the sclera, the muscle will be stretched and moved to a higher length-tension curve and affect motility to a degree.^[11,12]^ Beyond the range of normal elasticity, the extraocular muscles become stiff and act as a passive restraint. A crowding effect from a large bleb around a glaucoma implant with limited extraocular motility induces persistent diplopia.^[13,14]^ The limited movement in the direction of a glaucoma implant was also described in association with the Molteno implant and is related to a posterior fixation effect induced by scarring between the muscle belly and sclera behind the implant.^[15]^ Thus, the hypothesized cause of diplopia is mechanical disturbance attributable to the size of the plate, size of the resultant bleb and postoperative adhesion of the surrounding tissue of the rectus muscles.^[10–15]^ In the present case, because diplopia occurred the day after Baerveldt implantation and continued for 3 weeks, we speculated that the main causes were the mechanical disturbance induced by the large size of the plate and muscle contraction of the rectus muscles under which the Baerveldt was implanted rather than postoperative adhesion due to a scarring effect or temporal muscle edema.

The surface area of encapsulation around a glaucoma drainage implant is directly proportional to the end plate size. Therefore, the degree of IOP reduction achieved postoperatively is also directly proportional to implant size.^[16]^ Owing to the larger surface area of Baerveldt devices, they have become one of the most effective devices in controlling IOP. However, there appears to be an upper limit to plate size beyond which an increase in surface area may not improve pressure control and may even detrimentally affect surgical outcomes. There was no significant difference in surgical success and visual outcomes between the 350-mm^2^ and 500-mm^2^ Baerveldt glaucoma implants^[17]^ and between the 250-mm^2^ and 350-mm^2^ Baerveldt glaucoma implants^[18]^; additionally, there was a lower success rate with the 500-mm^2^ Baerveldt than with the 350-mm^2^ implant in a longer follow-up.^[6]^ Although the rate of strabismus after 2 types of Baerveldt implantation was not significantly different, that is, 16%^[17]^ and 20%^[6]^ in the Baerveldt 350-mm^2^ and 19%^[6,17]^ in the Baerveldt 500-mm^2^, or the rate of diplopia (2.7% in the Baerveldt 250-mm^2^ and 3.8% in the Baerveldt 350-mm^2^),^[18]^ Sun et al^[19]^ demonstrated that GDDs with a larger plate area (Baerveldt 350) had a higher frequency of diplopia (31%) than did GDDs with a smaller plate area (Baerveldt 250 or Ahmed; 11%), which was mainly attributed to the difference in implant size. Similarly, it was also speculated that the Ahmed 184-mm^2^ valve induced fewer motility disturbances than did the double-plate Molteno 270-mm^2^ or the Baerveldt 350-mm^2^ implant because of its smaller surface area.^[20–22]^ Currently, the Baerveldt 500-mm^2^ is not produced by the manufacturer based on the results of many clinical studies. The Baerveldt is now equipped with fenestrations in the end plate, allowing the growth of fibrous bands through the plate to reduce bleb height. Although the incidence of postoperative diplopia has been decreased by these modifications, diplopia remains a major complication that we must be aware of.

Finally, we reviewed the previous reports on treatment of diplopia due to glaucoma implant surgery. Smith et al^[7]^ reported that the removal of the Baerveldt 350-mm^2^ significantly improved diplopia in all 5 cases (replaced with double-plate Molteno implants in 4 cases and trabeculectomy after the removal in 1 case), but all the other interventions including botulinum injections in 1 case and prescription of prism glasses in 22 cases were only temporarily effective for diplopia and permanent diplopia did not resolve spontaneously in any of those cases. The authors used the old type of Baerveldt implant, which was not equipped with fenestrations in the end plate that allow the growth of fibrous bands through the plate to reduce bleb height; the use of this older implant could partially explain the lack of spontaneous resolution of diplopia. Muñoz and Parrish reported stable diplopia by observation in 4 small cases and commented on the difficulty of obtaining satisfactory results with prism glasses.^[11]^ These authors also mentioned that if strabismus surgery is performed, because it carries the risk of compromising a functioning drainage device by inducing scar tissue, the possibility of surgery in the fellow eye is often reluctantly accepted by the patient.^[11]^ Lloyd et al^[17]^ reported that although strabismus resolved in some eyes, 6 out of 13 patients underwent muscle surgery to correct motility dysfunction or diplopia. Sun et al^[19]^ reported that diplopia resolved in 1 and improved in 2 out of 11 post-GDD binocular diplopic patients by observation. The authors noted that the diplopia of these patients started immediately following tube opening and improved over 1 month.^[19]^ Roizen et al^[20]^ reported 7 patients with severe limitation to ocular rotations and incomitant strabismus who underwent strabismus surgery on an eye containing an implant and 2 patients with mild limitation to ocular rotations in the involved eye who underwent surgery on the contralateral eye. As a result, diplopia in the primary position was eliminated in 5 patients and markedly improved in 3 patients.^[20]^ The authors confirmed all patients had a large fibrous capsule surrounding the implant plate, adjacent muscles, and sclera; additionally, IOP was not elevated postoperatively in all cases.^[20]^ Rosenbaum and Santiago^[21]^ commented that nonsurgical conservative management consists of observation, prisms, and chemodenervation, but prisms are of limited use because of incomitance or the large angle of the deviation common in postoperative strabismus after GDD implantation. Moreover, the authors noted that surgical management includes strabismus surgery, removal of the implant and replacement of the implant with a smaller device.^[21]^ Additionally, in strabismus surgery, a muscle recession procedure is preferred over a muscle resection or muscle transposition procedure because of the reduced possibility of postoperative motor restriction or difficulty induced by the implant encroaching on the surgical field and sites of muscle attachment.^[21]^

This study has some limitations. Although there are no articles on the internet (PubMed, etc), plate revision, including its resection, could be a known intervention for postoperative diplopia following Baerveldt implantation to some clinicians. Even if this intervention is known to clinicians, our report is still worthwhile to the public as useful information that a glaucoma surgeon should know. In addition, the strabismus could have resolved without intervention, as it often does. However, no interventions could also lead to no improvement in postoperative strabismus. Thus, we should provide all possible treatment options to patients.

We reduced the size of the Baerveldt 350-mm^2^ by cutting and partially removing the plate, which was just beneath the rectus muscles. To the best of our knowledge, GDD size reduction surgery has not been previously reported. Although diplopia was relieved both subjectively and objectively, no drastic improvement was immediately observed, and we prescribed prism glasses for remaining mild diplopia during follow-up. However, 1 year after the operation, significant objective improvement was finally observed.

## 4 Conclusion

We reported a case of plate reduction surgery for a Baerveldt 350-mm^2^ glaucoma implant for postoperative motor disturbance. Early plate size reduction surgery, which was not immediately but ultimately effective in improving motor disturbance in our case, could be a potential option to relieve operation-induced motor disturbance. However, notably, tube shunt surgery has the risk of motility disturbances, which might require additional treatment.

## Acknowledgments

We thank all the staff of Tsukazaki Hospital who were involved in this study.

## Author contributions

**Conceptualization:** Hirotaka Tanabe, Shunsuke Nakakura.

**Investigation:** Hirotaka Tanabe, Shunsuke Nakakura.

**Project administration:** Hitoshi Tabuchi.

**Resources:** Shunsuke Nakakura, Asuka Noguchi.

**Supervision:** Hirotaka Tanabe, Shunsuke Nakakura, Yoshiaki Kiuchi.

**Validation:** Hirotaka Tanabe, Shunsuke Nakakura, Yoshiaki Kiuchi.

**Writing – original draft:** Hirotaka Tanabe.

**Writing – review & editing:** Hirotaka Tanabe, Shunsuke Nakakura, Yoshiaki Kiuchi.

Hirotaka Tanabe orcid: 0000-0002-1948-7408.
